# Shedding Light on Bladder Cancer Diagnosis in Urine

**DOI:** 10.3390/diagnostics10060383

**Published:** 2020-06-08

**Authors:** Kit Man Chan, Jonathan Gleadle, Jordan Li, Krasimir Vasilev, Melanie MacGregor

**Affiliations:** 1 School of Engineering, UniSA STEM, University of South Australia, Mawson Lakes, South Australia, SA 5095, Australia; kit_man.chan@mymail.unisa.edu.au; 2 Department of Renal Medicine, Flinders Medical Centre, Bedford Park, South Australia, SA 5042, Australia; jordan.li@sa.gov.au; 3 College of Medicine and Public Health, Flinders University, Bedford Park, South Australia, SA 5042, Australia; 4 Future Industries Institute, UniSA STEM, University of South Australia, Mawson Lakes, South Australia, SA 5095, Australia; krasimir.vasilev@unisa.edu.au

**Keywords:** bladder cancer, urine, noninvasive, 5-ALA, urinary biomarkers, photodynamic diagnosis

## Abstract

Blue light cystoscopy (BLC) is the most recent clinical approach in the detection and diagnosis of bladder cancer, a common type of cancer with a high rate of recurrence. Representing a significant advance over previous approaches, this photodynamic diagnostic technique uses a photosensitiser prodrug as an adjunct to white light cystoscopy to enhance the in vivo detection of malignant tissues in the bladder based on their distinctive fluorescence. Whilst it does improve detection rates, BLC remains an invasive and costly procedure. Meanwhile, a variety of noninvasive urine detection methods and related microdevices have been developed, none of which have yet entered routine clinical use due to unsatisfactory sensitivity. Following a brief description of the current approaches and their limitations, we provide here a systematic review of a newer niche research aiming to develop a noninvasive adaptation of photodynamic diagnosis. The research to date surrounding the ex situ use of photosensitiser prodrugs for urinary diagnosis of bladder cancer is also discussed.

## 1. Introduction

Urothelial bladder cancer (UBC) is the sixth most common cancer in the world. According to the World Health Organization (WHO), there were 549,000 new cases and 200,000 deaths from bladder cancer worldwide in 2018 [1]. Of particular concern is the fact that the commonest type of bladder tumour, nonmuscle invasive bladder cancer (NMIBC) has one of the highest recorded recurrence rates with up to 70% of patients experiencing at least one relapse [2]. Because of such alarming recurrence rates, long-term follow-up and monitoring are necessary. Guidelines from the European Association of Urology (EAU), the American Urological Association (AUA) and the Society of Urologic Oncology (SUO) advocate surveillance cystoscopy at 3–4 months after transurethral resection of bladder cancer in low-risk patients [3]. If the findings are negative, subsequent cystoscopy is advised after six to nine months, and then yearly for the next five years [3]. Such regular surveillance is time-consuming, expensive, uncomfortable and onerous.

There is currently no screening test for bladder cancer in asymptomatic people. Diagnostic procedures are performed in response to symptoms, with urine cytology and cystoscopy being the standard procedures. Urine cytology is a noninvasive method, based on the visual identification of abnormal cells from urine sediment following an extensive multistep sample preparation process. It has reasonable sensitivity for advanced tumours but for early or low-grade cancer, the sensitivity of cytology is only 33% [4]. Furthermore, the diagnosis is highly dependent on the specimen collection and the interpretation of the cytopathologist. White light cystoscopy (WLC) is an invasive procedure, in which an endoscope is inserted through the urethra for direct inspection of the bladder wall. A biopsy sample of the suspicious tissue is typically collected and analysed via histopathology for diagnosis and staging. This procedure is considered the “gold standard” for diagnosing bladder cancer. However, with the advent of blue light cystoscopy (BLC), which became FDA approved in 2010, it has been identified that the overall sensitivity of WLC for bladder cancer ranges from only 46% to 80% [5,6,7,8]. Additionally, WLC performs especially poorly in the detection of flat carcinomas, in particular tumour in situ (Tis), for which detection rates are lower than for Ta and T1 tumours [5]. BLC, also referred to as photodynamic diagnosis, exploits the photoactive properties of hexaminolevulinate (HAL), the ethyl ester derivative of 5-aminolevulinic acid (5-ALA). The exogenous administration of 5-ALA or its derivative lead to the preferential accumulation of fluorescent protoporphyrin IX in neoplastic tissues. Illuminated with blue-violet light, malignant tissue display a distinctive red fluorescence [9]. HAL-assisted BLC enhances the visual contrast between benign and malignant tissue which in turn improves diagnostic accuracy, particularly for NMIBC (defined as tumour stage T1, Ta or Tis), and patient outcomes [10,11]. Its long-term benefit with respect to cancer recurrence and progression remains unclear to date; nonetheless, both the AUA and EAU guidelines now recommend its use when available, particularly in patients with positive cytology and normal WLC [3,12]. As such, BLC represents one of this decade’s most significant advances in bladder cancer clinical management [13].

While the advantages of BLC over WLC are no longer contested [5,14], it remains an invasive technique. A noninvasive diagnostic and surveillance method with high sensitivity and specificity is needed to reduce the cost of surveillance and improve quality of life in people with bladder cancer. In the following section, we provide the current context for urinary biomarker testing, including commercially available tests and recent development involving microdevices, none of which has yet proven effective enough to supplant cystoscopy. We then conduct a systematic review of a new research axis where the photochemistry principle of BLC is applied to urine, working towards a new class of noninvasive diagnostic tests for urothelial cancer.

## 2. Current Noninvasive Test for Bladder Cancer Diagnosis Targeting Urinary Biomarkers

**Figure 1:**
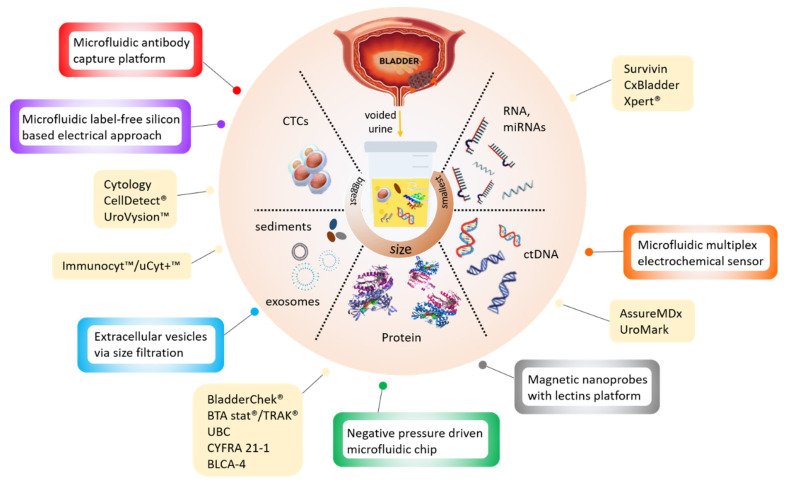
Infographic illustrating the currently available (yellow boxes) and potential microdevices (colour bordered boxes) for urinary bladder cancer diagnosis, as described in Table 1 and Table 2 below.

New noninvasive tests based on the detection of cancer-specific biomarkers in urine have been in development over the last decades. The different types of target biomarkers found in urine are summarised in Figure 1 below.

Table 1 provides a nonexhaustive list of studies involving these biomarkers that have resulted in commercially available tests for bladder cancer diagnosis. Most efforts have focused on detecting molecular biomarkers, i.e., tumour-specific proteins such as complement factor H-related protein, nuclear matrix protein (NMP) or UBC specific glycoproteins, primarily via immunochemical methods [15]. Several urine-based tests that detect these protein biomarkers have been commercialised and six of those approved by the FDA (BTA stat, BTA TRAK, NMP22, NMP22 BladderChek, uCyt+/ImmunoCyt and UroVysion) [16,17]. Other urinary tests under development that are not, to date, recommended for diagnostic use, include UBC-Rapid/ELISA test, CYFRA 21-1 and BLCA-1/BLCA-4, which assay proteins predominantly present in metastatic cells. These urine-based assays have the advantage of being noninvasive and rapid. They also have higher sensitivity than urine cytology but tend to be less specific and many suffer from variable performance [16,17,18] (Table 1). In addition, they have a lower sensitivity than white light cystoscopy for lower grade tumours (30–60%), with specificity ranging from 60% to 90%, and false-positive results in patients with inflammatory conditions [19]. The sensitivity and specificity values reported (Table 1) are highly dependent upon the clinical setting of the studies and discrepancy, therefore, arises from differences in patient cohort (selection criteria and size, tumour grades examined) and study design (primary or recurrent tumours; initial diagnosis or surveillance). Although some of these urine-based tests have been commercialised, their sensitivities and specificities have not been sufficient to justify changes in diagnostic or surveillance protocols. So far, the application of these new urine tests tends not to improve the identification of the disease but merely increase the associated costs [20].

In the quest for an accurate urinary biomarker for bladder cancer, many new—omics biomarkers have been reported [21,22], as recently summarised in comprehensive reviews [15,23,24]. Tests targeting genomic biomarkers that are commercially available are provided in Table 1. These tests typically detect DNA methylation, mutation or mRNA expression using PCR, SAGE and/or mass spectrometry methods. The detection of next-generation “omics” biomarkers may be more accurate but has the disadvantage of relying on expensive reagents and complex analytical platforms. 

In attempts to reduce the operating complexity of urinary tests without compromising their efficiency, existing (e.g., ELISA) and novel (e.g., cell membrane capacitance) detection approaches have been integrated into microdevices (Table 2). Most of these devices are still at a development stage and have not been rigorously assessed for clinical sensitivity and specificity. They target all types of bladder cancer biomarkers, including protein [34,35], DNA [36], extracellular vesicles [37,38] and whole cells [39,40,41] (Figure 1) but use advanced materials and nanotechnology to reduce analysis time and sample volumes.

For instance, a negative pressure-driven microchip integrating magnetic microbead-assisted immunocapture of bladder cancer biomarker apolipoprotein A1 (APOA1), report a measurement time of 40 min which is six times faster than a conventional ELISA test [34]. The detection of cancer-specific nucleic acid has been achieved using electrochemical impedance spectroscopy (EIS) and Surface plasmon resonance (SPR) within a microdevice containing porphyrin-tagged DNA probes [36]. Methods capable of detecting whole bladder cancer cells shed in urine are typically based on cell size, cellular features or the expression of specific proteins (e.g., intracellular galectin-1 or EpCAM). These microdevice-assisted approaches provide real-time detection in microliter volumes of urine [40] and reported specificity and sensitivity above 95% for the detection of cancer cells in spiked urine samples [39]. Microdevices provide an opportunity for the detection of novel biomarkers such as extracellular vesicles (EV) [42,43]. Tumour-derived EVs exist in various biological fluids, including urine, and carry cancer-specific proteins and nucleic acids. Technological approaches which capture and isolate bladder cancer EV through double-nanofiltration have been developed [37]. One of these approaches reported a sensitivity of 81% and a specificity of 90% in a modest cohort of 16 bladder cancer and eight healthy patients’ urine samples [38]. The advantages of these microfluidic devices over traditional EV isolation are that they require less processing steps and are, therefore, simpler and quicker (30 min). Furthermore, the final product contains nucleic acids and proteins that can be further used for genetic research which may provide personalised insight into the tumour heterogeneity. However, microdevice-based testing generally suffers from variations in the chemical and cellular composition of urine, as well as interpatient variability, more than conventional tests [44] because of the particularly low volume of the sample tested.

Overall, no urinary test based on urinary biomarker detection has yet replaced cystoscopy in screening and primary detection for NMIBC bladder cancer, according to current oncological guidelines (the American Urological Association (AUA)/Society of Urologic Oncology (SUO) [12], National Institute for Health and Care Excellence (NICE) [45], European Association of Urology (EAU) [46], and National Comprehensive Cancer Network (NCCN) [47]. Their use is not recommended for routine testing of low-risk NMIBC follow-up patients, and while they may be considered for the surveillance of high-risk NMIBC follow-up cases, the health care management plan for bladder cancer survivors still recommends including frequent cystoscopy and cytology. 

Despite these efforts, the greatest advance in bladder cancer diagnosis over the last decade remains blue light cystoscopy. A new body of work in the field has been identified where photodynamic diagnostic is used ex vivo in combination with microdevices. We will now review research which capitalises on the fundamental principle underpinning the cancer specificity of blue light cystoscopy to develop photobiology-enabled tests for noninvasive bladder cancer diagnostics.

## 3. Current Use of Photodynamic Diagnostic for Bladder Cancer Detection

**Figure 2:**
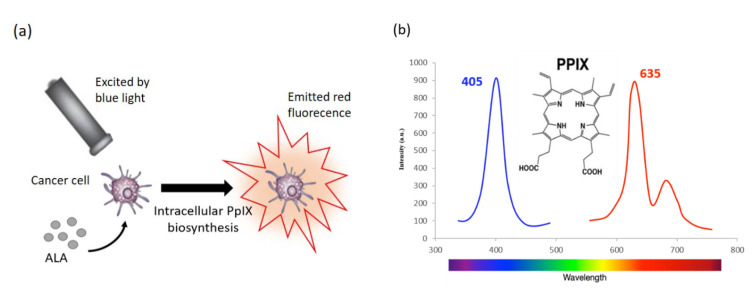
The principle of photodynamic diagnosis (PDD) of 5-ALA mediated protoporphyrin IX (PpIX) in tumour cells (**a**); molecular structure of PpIX and fluorescence spectrum (**b**), following excitation with violet blue light at 405 nm, PpIX emits a red fluorescence of 635 nm.

Blue light fluorescent cystoscopy using 5-aminolevulinic acid (5-ALA) as a photosensitiser prodrug has been introduced into practice in several countries, following its FDA approval for intraoperative PDD of bladder cancers in 1999 [48]. 5-ALA is a naturally occurring amino acid, a precursor in the biosynthetic pathway for heme and porphyrin production. Numerous studies [49,50,51,52] have identified that exogenous administration of 5-ALA leads to the preferential accumulation of endogenous protoporphyrin IX (PpIX) in tumour cells when compared with normal, healthy cells. Specifically, Krigemer et al. found that, following exogenous administration of 5-ALA, PpIX accumulates 17-fold more in urothelial neoplastic lesions than normal cells [49]. Though the mechanism for this cancer-specific accumulation is incompletely understood [53]. PpIX is a photosensitiser and, when excited by blue light, it displays red fluorescence (Figure 2).

**Figure 3:**
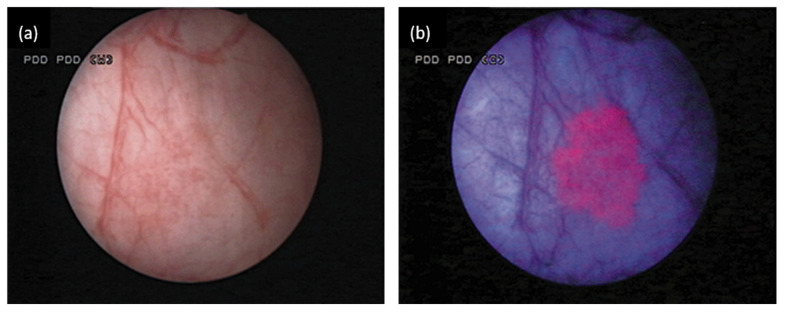
White light (**a**) and blue light fluorescence (**b**) endoscopic image of a flat tumour in situ (Tis). 5-ALA solution was instilled intravesically before cystoscopy. This is revealed by reddish fluorescence when using fluorescence cystoscopy. Reproduced with permission of John Wiley and Sons on behalf of the British Journal of Urology (BJU) International [50].

The preferential accumulation of the photosensitiser PpIX in tumour tissue means that bladder cancer tissues show brighter and sharper red fluorescence than normal tissues when excited with blue-violet light [50]. This is particularly helpful in the diagnosis of high-grade flat lesions like carcinoma in situ (CIS) which are missed easily with WLC (Figure 3). Inoue et al. [52] compared the diagnostic accuracy between 5-ALA-assisted BLC and WLC in primary and recurrent bladder cancer cases. They concluded that BLC can significantly increase bladder tumour detection rate to over 80% sensitivity when compared with conventional cystoscopy [51,52]. This fluorescence intensity difference between tumour and normal cells has since been recognised as a useful diagnostic tool in cancer detection in vivo.

5-ALA, however, has limited bioavailability because it is a polar charged zwitterion. Esterification of 5-ALA has been used to improves its lipophilicity, thus overcoming the challenge of low bioavailability. Hexyl ester (hexyl aminolevulinate or hexaminolevulinate (HAL)) is the most successful 5-ALA derivative, and has been approved by the FDA and the European Association of Urology for the photodetection of bladder cancer via fluorescently assisted cystoscopy in 2010. HAL induces higher PpIX fluorescence at lower concentrations and with shorter time-spans. HAL-induced fluorescence cystoscopy improves the effectiveness of PDD in bladder cancer diagnosis [5].

**Figure 4:**
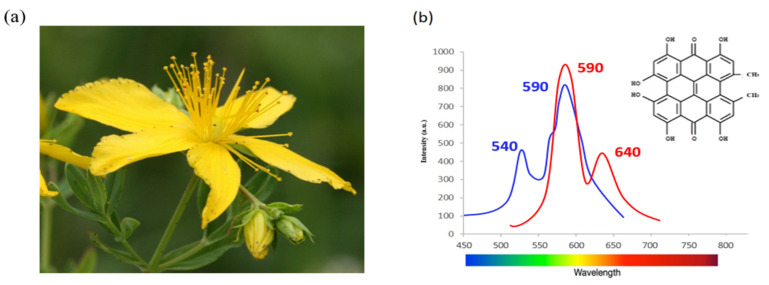
St. John’s wort (**a**). Reproduced with permission of photographer [55]; molecular structure of hypericin and the fluorescence spectrum of hypericin (**b**).

Another important shortcoming of 5-ALA and its hexyl derivative is their fast photobleaching. For example, Sharwani et al. [54] reported that the sensitivity of the fluorescence detection system was strongly affected by the photobleaching rate. An alternative potent natural photosensitiser with higher resistance to light exposure, Hypericin, has been investigated for PDD application. Hypericin is an extract from the flowering plant *Hypericum perforatum* also called St. John’s Wort. It is a polycyclic fluorescent red pigment present in different parts of the plant. Hypericin has absorbance maxima at 540 and 590 nm and emits an orange-red fluorescence at 590 nm and 640 nm as shown in Figure 4.

The light emission of hypericin is strongly oxygen-dependent, hypericin generally shows minimal or no toxicity in the dark but generates singlet oxygen and reactive oxygen species (ROS) with a light wavelength around 590 nm [56]. These radicals are cytotoxic and can kill cells by apoptosis and/or necrosis depending on dosage. This photosensitising ability of hypericin has been of interest as a diagnostic tool for PDD of flat bladder carcinoma in situ. After intravesical instillation, hypericin accumulates selectively in tumour tissues and displays red fluorescence using fluorescence endoscopy [57]. HY has an excellent tumour specificity rate in vivo [57,58] with less photobleaching problems than 5-ALA. 

The drawbacks of HY, however, are low solubility, costly production and hydrophobicity in solution. For these reasons, 5-ALA remains the most popular photosensitiser prodrug in clinical settings, however, the high resistance to photobleaching displayed by hypericin means that this alternative photosensitiser could be promising for ex situ applications, where light exposure is more of an issue.

## 4. Use of Photosensitiser Prodrug in Urine Towards Noninvasive Diagnostic

**Figure 5:**
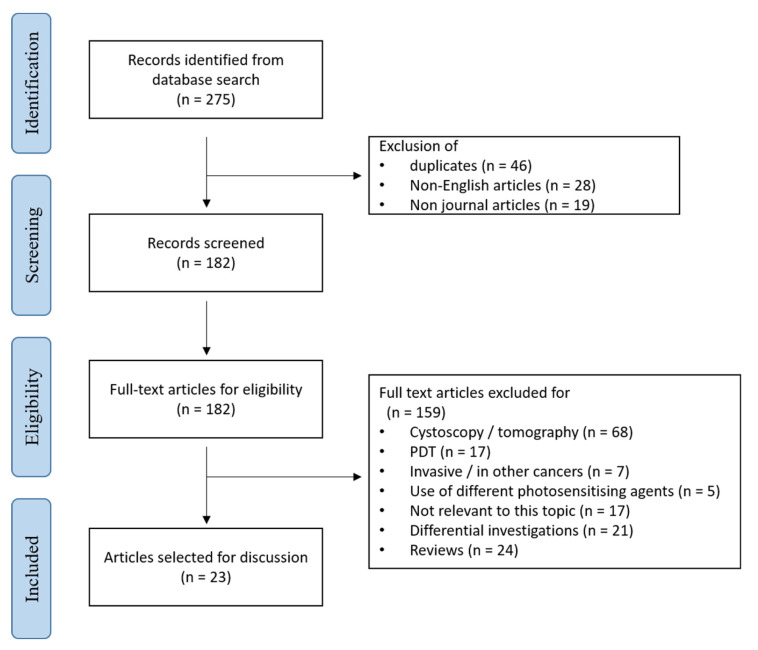
Flow diagram of evidence acquisition.

A systematic search of the literature published between 1950 and 2020 was conducted using PubMed and Web of Science. The search strategy used the following keywords: (bladder cancer or transitional cell carcinoma or urothelial carcinoma) and (hexaminolevulinate or aminolevulinic acid or protoporphyrin IX or 5-aminolevulinic acid or hypericin) and (detection or diagnosis) and (Urinary cytology or urine or noninvasive) and (fluorescence or photodynamic). Based on these initial selection criteria, a total of 275 original articles were identified. 46 duplicates, 28 articles not written in English and 19 articles not belonging to journal articles were excluded. Papers were eligible for inclusion where they focus on an ex vivo or in vitro use of 5-ALA, its derivative or hypericin for diagnostic purpose. Reports on invasive diagnostic methods, specifically blue light cystoscopy, photodynamic therapy (PDT) or the use of different photosensitising agents were, for instance, excluded. Following screening for eligibility, 23 studies met the criteria (Figure 5) [59,60,61,62,63,64,65,66,67,68,69,70,71,72,73,74,75,76,77,78,79,80,81,82]. Nineteen publications referred to the use of 5-ALA and its derivative as photosensitiser prodrugs, and four on that of hypericin as photosensitisers. The primary outcome from the cell lines studies was the utility of ex vivo PDD (Table 3). Photosensitiser-induced fluorescent urine cytology (Table 4) was the secondary endpoint we identified.

### 4.1. 5-ALA-based Approaches

From the mid-1990s, several studies have investigated the response of cell lines to the exogenous administration of 5-ALA [59,60,61,62]. These in vitro studies analysed 5-ALA incubation conditions and its phototoxicity in order to define and optimise the practical procedure to be used in BLC and PDT. Those early studies informed nowadays in vivo clinical practices (Table 3). We have recently conducted a comparable study using HAL as the photosensitiser prodrug and identified incubation conditions where bladder cancer cells display fluorescence intensity up to four-fold higher than control healthy cells [71].

The combination of 5-ALA intravesical instillation followed by urinary cytology later emerged as fluorescence cytology [63,64,65], an ex vivo approach for the diagnosis of bladder cancer which still required the invasive instillation of 5-ALA into the bladder. Table 4 summarises all studies involving patient samples for the use of photosensitiser prodrug in ex vivo diagnostics. The idea of combining the principle of photodiagnosis and urinary cytology was first proposed by Pytel and Schmeller in 2002 [63]. This initial approach tested the clinical relevance of both 5-ALA as photosensitiser prodrugs and hypericin as photosensitisers instilled directly into the patient’s bladder through a catheter prior to transurethral resection of the bladder tumour (TURBT). Urine samples were collected for fluorescence microscopy after a minimum of 1h intravesical retention of the photosensitiser prodrugs. Tauber et al. began investigating the feasibility of 5-ALA-induced fluorescence cytology in human bladder washes in 2003 [64] using a similar approach. Later they developed a differential spectrophotometric method for 5-ALA-assisted tumour cell detection in bladder lavage fluid. However, many samples could not be reliably analysed using this technique because the signal from urothelial cells was often too low when associated with many interfering red blood cells [65]. Despite the promising results obtained from fluorescent cytology, the instillation procedure remained invasive.

5-ALA administration was investigated by Inoue’s group. 5-ALA was orally administered to 66 bladder cancer patients and 20 healthy volunteers, urinary porphyrin concentrations were measured by high-performance liquid chromatography (HPLC). They indicated that the fluorescence porphyrins can act as tumour biomarkers in urine samples [66], which can be integrated into a noninvasive detection approach.

Since then, the ex vivo administration of 5-ALA [67,69] and HAL [73] or the noninvasive photodynamic diagnostic of bladder cancer in urine has been evaluated in six studies, three of which were undertaken by Fujimoto’s research group (Table 4).

They conducted several experimental studies to refine the use of 5-ALA for ex vivo PDD where the photosensitiser prodrug is added directly to voided urine samples. In a comparative study [67], urine from 58 bladder cancer patients was examined via urinalysis, cytology and urinary marker tests (BTA and NMP22 tests). High-grade bladder cancer T24 cells and the urine samples collected from 20 benign patients were used as positive and assay controls, respectively. The urine samples and the T24 cells were centrifuged and the cell pellet was resuspended with 1 mM 5-ALA, incubated for 2 h. Cellular fluorescence following 5-ALA exposure was detected by fluorescence microscopy, microplate spectrophotometer and their own device “cellular fluorescence analysis unit”, CFAU. The overall sensitivity of the 5-ALA fluorescence detection methods was high (≥ 86%), for both low-grade and high-grade tumours. Whereas the sensitivity of BTA and NMP22 was only 33% and 40%, respectively. A further study used ALA-induced PpIX levels to detect exfoliated tumour cells in voided urine samples via simple spectrophotometry [68]. The sensitivity of ALA-induced fluorescence cytology (82%) was higher than conventional cytology (49%) in low-grade tumours and NMIB tumours. Recent studies by Yamamichi et al. [69,81] compared 5-ALA guided fluorescent urine cytology to conventional cytology for bladder cancer detection in patient urine samples. They found overall sensitivity and similar specificity rates as the Fujimoto group (5-ALA vs. conventional urine cytology: sensitivity 86.9% to 90.4% vs. 66.3% to 69.4% and specificity 96.2% to 100% vs. 95.6% to 98.2% respectively). These studies also demonstrated the higher sensitivity for 5-ALA-based PDD over conventional cytology in pT1 stage and below (5-ALA vs. conventional urine cytology: sensitivity 89.1% to 91.4% vs. 67.3% to 69%) and low-grade tumour (5-ALA vs. conventional urine cytology: sensitivity 88.5% to 91.5% vs. 51.1% to 53.8%). Together, these results support the principle of use of ALA-based PDD assay from urine samples as an alternative detection method for bladder cancer. The Fujimoto group has since explored ways to extend the use of 5-ALA to the urinary PDD of prostate cancer [72,77].

Attempts to improve the performance of this method have included the use of HAL as the fluorescence inducing agent. HAL may be a better candidate than 5-ALA for ex vivo use in urine samples because it induces the intracellular PpIX accumulation at lower concentration and shorter incubation time than 5-ALA in vitro [83]. Furthermore, HAL, unlike 5-ALA, does not induce any porphyrin fluorescence in urinary bacteria [84]. Using HAL may, therefore, reduce the chance of false-positive results and increase the potential specificity of the tests. Yet, reports investigating the usefulness of HAL for the noninvasive diagnosis of bladder cancer are sparse and only three studies [70,71,73] have been published to date. Čunderlíková and colleagues examined the rate of HAL-induced PpIX production by flow cytometry in normal urinary bladder transitional epithelial cells (NBECs) from healthy volunteer urine and in the human bladder transitional carcinoma cell line (TCCSUP). This original work did demonstrate the potential of HAL-induced fluorescence for the detection of cancer cells in urine samples [70]. The Fujimoto group reported that using HAL improved the detection of bladder cancer cells in urine samples compared to 5-ALA [73]. More recently, our findings also showed that HAL-induced PpIX fluorescence was significantly high in bladder cancer cells compared to noncancer cells [71]. 

### 4.2. Hypericin-Based Approaches

Although hypericin has a strong tumour selectivity in vivo, an in vitro study investigating the hypericin-induced photosensitivities of different cultured cell monolayers (human bladder cancer and nonbladder cancer) revealed that normal cells displayed the same photosensitivity as cancer cells [85]. While little is known about the mechanism responsible for the selective accumulation of hypericin into tumour tissue, the absence of selective hypericin uptake in cancer cells in vitro seems to indicate that the answer may lie in elements of the tumour microenvironment. The correlation of hypericin cellular uptake and accumulation has been studied using multicellular tumour spheroids within a three-dimensional (3D) matrix, with the aim to mimic the tumour biological features in vitro. These tumour models provide an insight into cell-to-cell interactions and pharmacodynamic responses, and have provided evidence showing that the adhesion molecules in tumour cells such as cadherins play a critical role [86] for hypericin tumour selectivity. 

Nonetheless, following Pytel and Schmeller initial work with hypericin instillation [63], Olivo and colleagues [76] indicated the possibility of using hypericin ex vivo for early bladder cancer screening. Twenty-nine urine samples from healthy volunteers and from patients who were suspected of having early bladder cancer were processed and centrifuged. The pellet was resuspended in hypericin (1 mg/mL in dimethyl sulphoxide DMSO) and incubated for 15 min at room temperature. Cell pellets obtained after another centrifugation step were observed via confocal microscopy. The fluorescence intensity of low-grade urothelial tumour cells was 8.5 times stronger than normal cells after the hypericin incubation. This differential was deemed large enough to allow for an automated quantitative analysis of the emitted fluorescence. In a follow-up, ex vivo urine fluorescence cytology study [74], a diagnostic algorithm was described to discriminate between normal and low-grade lesions using both sell size and hypericin-induced fluorescence. While these studies indicated that hypericin could constitute a selective tumour marker for early bladder cancer detection, there has been no further report published on this topic since 2007. 

## 5. Conclusions

5-ALA guided cystoscopy improves bladder cancer detection sensitivity compared with WLC but remains a costly invasive procedure. In contrast, cytology is noninvasive and selective but it has limited sensitivity. The application of cancer-specific photosensitiser prodrugs in urine cytology has the potential to combine the strength of both BLC and conventional cytology. This approach would also be a cost-effective alternative to next-generation omics-based tests, which could, therefore, be used for routine surveillance testing. 

However, in bringing bladder cancer detection techniques towards routine clinical use a number of challenges remain. Using fluorescence detection techniques in isolation is still associated with a significant number of false-positive tests and the low number of cancer cells shed in urine in many patients is a clear limitation. The use of immune-assisted microfluidic devices designed to enrich the cancer cells population compared to the background benign cells might overcome these issues and combined with HAL and/or hypericin may have the potential for improving bladder cancer diagnosis from urine samples.

## Figures and Tables

**Table 1:** Summary of available tests based on the detection of urinary biomarkers [16,17]. A non-systematic literature research was performed using the PubMed/Medline database. Searched by using the following keywords: “bladder cancer”, “urinary markers”, “biomarkers”, “diagnosis”, “detection”, “urine biomarkers”, “NMIBC”, “surveillance”. The search was conducted in 2020.

Test (Manufacturer)	Detected Biomarker	Assay type	Sensitivity %	Specificity %	Development Stage *	FDA Approved	Ref.
Urine Cytology	Atypical urothelial cells	Microscopy	33.3	100	Clinical practice	NA	[4]
NMP22/BladderChek^®^ (Abbott Laboratories, IL, USA)	Nuclear mitotic apparatus proteins (Nuclear matrix protein-22)	Sandwich ELISA/point-of-care test	33–77	75–97	FDA approved diagnosis and follow-up	1996/2002	[18]
uCyt+™/Immunocyt™ (Scimedx Corporation, NJ, USA)	Bladder tumour cell associated mucins/carcinoembryonic antigen (antibodies19A211, LDQ10 and M344)	Immunocytochemistry	78–90	77–87	FDA approved follow-up	2000	[18]
UroVysion ™ (Abbott Laboratories, IL, USA)	Aneuploidy and loss of loci (chromosomes 3, 7, 17 and 9p21 loci)	Multicoloured and multiprobed FISH	50–88	87–98	FDA approved diagnosis and follow-up	2002	[18]
BTA stat^®^/TRAK^®^ (Polymedco Inc., NY, USA)	Complement factor H-related protein	Dipstick immunoassay/sandwich ELISA	61–87	38–87	FDA approved diagnosis and follow-up	1998/1997	[18]
UBC-Rapid/ELISA test (IDL Biotech AB, Bromma, Sweden)	Cytoskeletal protein (cytokeratin 8 and 18)	Sandwich ELISA/point-of-care test	48.7–70.5	64.5–79.3	Clinical laboratory research		[4]
CYFRA 21-1 (Roche Diagnostics, IN, USA)	Cytoskeletal protein (cytokeratin 19)	Electrochemiluminescent immunoassay/ELISA/immunoradiometric assay	82	80	Clinical laboratory research		[25]
BLCA-4 (Eichrom Technologies, IL, USA)	Nuclear matrix protein (BLCA-4)	Sandwich ELISA	96.4	100	Clinical laboratory research		[26]
Survivin (Fujirebio Diagnostics Inc., PA, USA)	Inhibitor of apoptosis gene	Bio-dot test	64	93	Clinical trial		[27]
Cx Bladder (Pacific Edge Diagnostics, PA, USA)	mRNA expression of genes (IGF, HOXA, MDK, CDC and IL8R)	RT-qPCR	91	96	Clinical trial		[28]
AssureMDx (MDxHealth, CA, USA)	Methylation analysis (OTX1, ONECUT2 and TWIST)/mutation analysis (FGFR3, TERT and HRAS)	Methylation/mutation analysis	57–83	59	Clinical laboratory research		[29]
Xpert^®^ bladder cancer monitor (Cepheid Inc., CA, USA)	mRNA expression of genes (CRH, IGF2, UPK1B, ANXA10 and ABL1)	RT-qPCR	73	77–90	Clinical trial		[30]
UroMark (Kelly:Feber Lab, UCL, UK)	Targeted loci DNA methylation (150 CpG loci)	Microdroplet-based PCR and NGS	98	97	Clinical trial		[31]
CellDetect ^®^ (Micromedic Technologies Ltd., Tel Aviv, Israel)	Atypical urothelial cells	Microscopy	94	89	Clinical trial		[32]

* According to the ClinicalTrials.gov, a source provided by the U.S. National Library of Medicine [33]. Development stages are considered as “Clinical laboratory research” and “Clinical trial”. ELISA: enzyme-linked immunosorbent assay; UBC: urinary bladder cancer; RT-qPCR: reverse transcription quantitative polymerase chain reaction; NGS: next-generation sequencing.

**Table 2:** Types of microdevices for bladder cancer detection in urine.

Microdevices	Detected Marker	Assay Type	Ref.
Negative pressure-driven microfluidic chip	APOA1 protein via antibody capture on magnetic microbead	ELISA	[34]
Magnetic nanoprobes with lectins platform	Glycoproteins via Glycoproteomics and CD44 expression	Slot-blot analysis, immunohistochemistry	[35]
Microfluidic multiplex electrochemical sensor	cfDNA via DNA hairpins bound to electrode, DNA methylation	SPR/EIS	[36]
Microfluidic antibody capture platform	Cancer cell capture via EpCAM on POx coating	Point-of-care test	[39]
Antibody conjugated nanoprobes immunosensor	Intracellular Gal-1 protein via immunosensor	Point-of-care test	[40]
Microfluidic label-free silicon-based electrical approach	Whole cells via membrane capacitance difference	Flow cytometry	[41]
Microfluidic double filtration	Extracellular vesicles via size filtration	ELISA	[37,38]

APOA1: apolipoprotein 1; SPR: surface plasmon resonance; EIS: electrochemical impedance spectroscopy; cfDNA: cell-free deoxyribonucleic acid; EVs: extracellular vesicles; EpCAM: epithelial cell adhesion molecule; Gal-1: galectin-1; POx: polyoxazoline.

**Table 3:** Studies of prodrug application in cell lines or in vivo.

Study (Year)	Prodrug	Types of Samples Tested	Methodological Approach *	Ref.
Steinbach et al. (1995)	5-ALA	In vitro ● Human urothelial cancer J82 and RT4 cells ● Human normal urothelial HCV29 cells ● Human umbilical cord endothelial cells ● Human fibroblasts	30–300 µg/mL 5-ALA in serum-containing medium for 4 h at 37 °C	[59]
Riesenberg et al. (1996)	5-ALA	In vitro ● Human urothelial cancer J82 and RT4 cells ● Human normal urothelial HCV29 cells	25–100 µg/mL 5-ALA in serum-containing RPMI 1640 medium for 4 h at 37 °C	[60]
Li et al. (1999) [61]	5-ALA	In vivo ● Balb/c mice with fibrosarcoma In vitro ● Mouse normal fibroblasts ● Mouse fibrosarcoma cells ● Human bladder carcinoma T24 cells ● Human lung cancer A549 cells	In vivo ●100–300 mg 5-ALA per kg of body weight, injected intraperitoneally for 3 h In vitro ●3mM 5-ALA in RPMI-1640 medium with 1% FCS for 15 h at 37 °C	[61]
Krieg et al. (2000)	5-ALA	In vitro ● Human urothelial cancer J82 and RT4 cells ● Human normal urothelial UROtsa cells ● Human fibroblasts	10–200 µg/mL 5-ALA in serum-free medium for 3 h at 37 °C	[78]
Seidl et al. (2001)	5-ALA	In vitro ● Human urothelial cancer RT4 cells ● Human normal urothelial UROtsa cells	100 and 200 µg/mL 5-ALA in serum-free medium for 3 and 1 h at 37 °C	[62]
Inoue et al. (2009)	5-ALA	In vitro ● Human urothelial cancer 253J-P, 253J-BV, T24 cells ● Human prostate carcinoma PC-3 and DU145 cells ● Human renal carcinoma ACHN, 786-o and A-498 cells ● Human renal epithelial RPTEC cells	0–1 mM 5-ALA in serum-free medium for 0-6 h at 37 °C	[80]
Chan et al. (2019)	HAL	Ex vivo ● Trypsinized cells (human bladder cancer HT1197 and HT1376; human fibroblast HFF) in suspension	10–150 µM HAL in serum-free medium for 30 min to 6 h at 37 °C	[71]
Shirazi et al. (2020)	HAL	Ex vivo ● Trypsinized cells (human prostate LNCaP; human normal prostate epithelium PNT2) in suspension	50–150 µM HAL in PBS for 30 min to 2 h at 4, 23 and 37 °C	[82]
Crnolatac (2005)	Hypericin and its derivatives	In vitro ● Human bladder carcinoma RT112 cells	1 µM hypericin/hypericin derivatives in MEM for 2 h at 37 °C	[75]

* only the conditions for 5-ALA- or HAL-induced PpIX fluorescence measurements were noted. ALA: aminolevulinic acid; HAL: hexyl aminolevulinate or hexaminolevulinate; MAL: methyl aminolevulinate; MEM: minimum essential medium.

**Table 4:** Studies of prodrug application ex vivo.

Study (year)	Prodrug	Cohort Size	Methods	Administration	Specimen	Ref.
Pytel et al. (2002)	5-ALA	38 ^a^	1.5 g 5-ALA in 50 mL of 1.5% sodium bicarbonate for > 1 h	Intravesical instillation	Urine	[63]
Tauber et al. (2003)	5-ALA	162 ^a^	50 mL of 3% 5-ALA buffered with sodium monohydrogen-phosphate for 2 h	Intravesical instillation	Lavage solution	[64]
Tauber et al. (2006)	5-ALA	62 ^a^	50 mL of 3% 5-ALA buffered with sodium monohydrogen-phosphate for 1 to 2 h	Intravesical instillation	Lavage solution/sediments	[65]
Inoue et al. (2013)	5-ALA	66 ^a^	1.0 g 5-ALA	Oral	Urine	[66]
Miyake et al. (2014)	5-ALA	58 ^a^	1 mM of 5-ALA in serum-free RPMI-1640 medium for 2 h at 37 °C	Exogenous 5-ALA added to urine pellet	Urine	[67]
Nakai et al. (2015)	5-ALA	61 ^a^	1 mM of 5-ALA in PBS for 2 h at 37 °C	Exogenous 5-ALA added to urine pellet	Urine	[68]
Nakai et al. (2017)	5-ALA	50 ^a^	1 mM of 5-ALA in PBS for 2 h at 37 °C	Exogenous 5-ALA added to urine pellet	Urine	[73]
Yamamichi (2019)	5-ALA	160 ^a^	200 µg/mL 5-ALA in MEM for 2 h at 37 °C	Exogenous 5-ALA added to urine pellet	Urine	[69]
Yamamichi (2020)	5-ALA	104 ^a^	1.2 mM 5-ALA in MEM for 2 h at 37 °C	Exogenous 5-ALA added to urine pellet	Urine	[81]
Čunderlíková et al. (2007)	HAL	19 ^a^	50 µM HAL in serum-free RPMI-1640 medium for 1 h	Exogenous HAL added to urine pellet	Urine	[70]
Nakai et al. (2017)	HAL	50 ^a^	0.1 mM HAL in PBS for 2 h at 37 °C	Exogenous HAL added to urine pellet	Urine	[73]
Nakai et al. (2014)	5-ALA	138 ^b^	1 mM of 5-ALA in PBS for 2 h at 37 °C	Exogenous 5-ALA added to urine pellet	Urine	[72]
Nakai et al. (2018)	5-ALA	189 ^b^	1 mM of 5-ALA in PBS for 2 h at 37 °C	Exogenous 5-ALA added to urine pellet	Urine	[77]
Pytel et al. (2002)	Hypericin	8 ^a^	40 mL of an 8-µmol/L solution of hypericin for > 1 h	Intravesical instillation	Urine	[63]
Olivo et al. (2003)	Hypericin	29 ^a^	1 mL of hypericin in serum-free RPMI-1640 medium for 15 min at room temperature	Exogenous hypericin added to urine pellet	Urine	[76]
Fu et al. (2007)	Hypericin	21 ^a^	1 mL of hypericin in serum-free RPMI-1640 medium for 15 min at room temperature	Exogenous hypericin added to urine pellet	Urine	[74]

^a^ urothelial carcinoma patient; ^b^ prostate cancer patient. ALA: aminolevulinic acid; HAL: hexyl aminolevulinate or hexaminolevulinate; MEM: minimum essential medium.
